# Designed Elastic Networks: Models of Complex Protein Machinery

**DOI:** 10.3390/ijms19103152

**Published:** 2018-10-13

**Authors:** Holger Flechsig, Yuichi Togashi

**Affiliations:** 1 Nano Life Science Institute (WPI-NanoLSI), Kanazawa University, Kakuma-machi, Kanazawa, Ishikawa 920-1192, Japan; 2 Department of Mathematical and Life Sciences, Graduate School of Science, Hiroshima University, 1-3-1 Kagamiyama, Higashi-Hiroshima, Hiroshima 739-8526, Japan; togashi@hiroshima-u.ac.jp

**Keywords:** mechanical networks, evolution, design, protein machines, molecular motors, Brownian ratchet, power stroke, allostery, conformational motions, synthetic protein

## Abstract

Recently, the design of mechanical networks with protein-inspired responses has become increasingly popular. Here, we review contributions which were motivated by studies of protein dynamics employing coarse-grained elastic network models. First, the concept of evolutionary optimization that we developed to design network structures which execute prescribed tasks is explained. We then review what presumably marks the origin of the idea to design complex functional networks which encode protein-inspired behavior, namely the design of an elastic network structure which emulates the cycles of ATP-powered conformational motion in protein machines. Two recent applications are reviewed. First, the construction of a model molecular motor, whose operation incorporates both the tight coupling power stroke as well as the loose coupling Brownian ratchet mechanism, is discussed. Second, the evolutionary design of network structures which encode optimal long-range communication between remote sites and represent mechanical models of allosteric proteins is presented. We discuss the prospects of designed protein-mimicking elastic networks as model systems to elucidate the design principles and functional signatures underlying the operation of complex protein machinery.

## 1. Introduction

Most dynamical processes in biological cells involve proteins which operate as molecular machines and motors. They represent engines which, at the nanoscale, convert chemical energy utilized from ATP hydrolysis into mechanical deformations of their structure. Perfected through biological evolution, each machine is therefore able to execute its specific function as part of a complex factory of macromolecules to establish life at the nanoscale [1,2].

In theoretical descriptions of protein machines and motors, there is a gap between detailed, structure-based, molecular dynamics approaches and oversimplified phenomenological models. While, on the one hand, all-atom simulations can capture only a fraction of the relatively slow full operation cycles despite the use of super-computers, on the other hand, drastically reduced models typically lack any structural resolution. Hence, fundamentally important aspects underlying, e.g., intramolecular communication and allosteric coupling in molecular machines, or the mechanism of force transduction in protein motors, are still not sufficiently understood.

To bridge this gap, models of intermediate complexity, such as the coarse-grained elastic network (EN) model of proteins, have been widely used. A protein elastic network is constructed as a set of beads connected by elastic springs [3,4,5]. Each bead typically corresponds to a single amino acid residue, and elastic connections mediate effective interactions between beads. Despite obvious simplifications, such models can reproduce conformational fluctuations in proteins, and, remarkably, normal modes of elastic networks have been shown to be able to describe even the large-amplitude motions related to ATP binding and hydrolysis in various molecular machines and motors (e.g., [6,7,8]). Moreover, in several previous studies, protein elastic networks have been considered as dynamical systems, and conformational dynamics in response to external forcing has been probed in order to investigate functional mechanochemical motions in protein machines [9,10,11,12,13,14,15,16,17].

Recently, a new perspective on mechanical networks with elastic interactions has been popularized, with applications ranging from material sciences to biophysical problems. Inspired by the behavior found in proteins, the computational design of network structures which execute specific prescribed tasks and the control of their functional properties have become en vogue. Several groups have successfully designed networks which, inspired by allosteric effects in proteins, reveal pronounced communication between spatially remote regions, and their functional properties have been analyzed [18,19,20,21].

In this topical review, we summarize our contributions to this exciting and emerging field with emphasis placed on the perspective of the study of protein dynamics employing physics-based coarse-grained elastic networks. Starting with an explanation of the concept of evolutionary optimization, we present what we believe marks the origin of the idea to design complex mechanical networks with protein-inspired responses, namely, the design of an elastic network structure which mimics the cycles of ATP-powered conformational motions in protein machines [9]. We then proceed with two recent applications. First, the construction of a model molecular motor is reviewed. It mimics the operation of muscle myosin, and its function incorporates both the tight coupling power stroke as well as the loose coupling Brownian ratchet mechanism [22]. The second application concerns the evolutionary design of elastic networks which encode optimized long-range communication and represent coarse-grained model structures of artificial allosteric proteins [20].

We also provide a discussion on the prospects of designed functional networks as structurally resolved model systems to better understand the design and functional principles of complex protein machines.

## 2. Design of Functional Elastic Networks

The concept of designing elastic networks which have desired properties was introduced by Togashi and Mikhailov in their 2007 work [9]. They designed elastic networks which were able to conduct collective and ordered conformational motions. One designed example network, in which the intrinsic soft motion consisted of well-defined relative movement of two globular domains, was then used to construct a model of a molecular machine. The large-amplitude soft motion was excited by the binding and unbinding of a fictitious ligand in the flexible hinge region connecting the two domains. Thus, a model machine which was able to perform ordered cyclic internal motions powered by ligands could be obtained, and it was further demonstrated that its operation was robust, even in the presence of thermal fluctuations.

Recently, we used this mimic of a protein machine to construct a model system of a molecular motor with an operation mechanism resembling that of the muscle myosin motor protein [22]. It consists of the elastic machine and a rod-like filament with which it is allowed to interact. Through binding and unbinding the filament in a ratchet-like fashion, the machine is able to convert its cyclic conformational motions effectively into steady translocation of the filament. By performing extensive numerical simulations of this structurally resolved model, we demonstrated that this artificial motor assembly can operate similarly to a deterministic, or Brownian, ratchet, thus incorporating both scenarios that are commonly employed by the community to characterize the modus operandi of molecular motors.

We recently established structurally resolved model systems of allosteric proteins by considering the design of elastic network structures which encode optimal long-range communication between distant sites [20].

Before we review the applications, we first explain the concept of computational evolution which was developed to design elastic networks with prescribed protein-inspired functions.

## 3. Evolutionary Optimization

**Figure 1:**
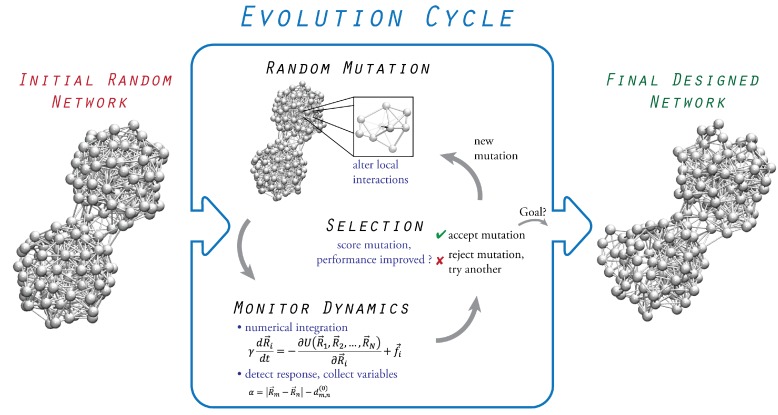
Design scheme of evolutionary optimization. Starting from an inactive random elastic network, functional elastic networks are designed within a cycle of in silico evolution. A sequence of mutation followed by selection is iteratively applied to the network to improve its performance towards the intended prescribed task.

The design process relies on the evolution of initially inactive structures which alter their architecture and pattern of physical interactions to improve and eventually optimize their performance towards a prescribed functional task. The full evolution scheme is shown in Figure 1, and the involved steps are described in the following text.

### 3.1. Initial Random Network

When elastic networks of proteins are constructed, the respective experimentally determined structure deposited in the Protein Data Bank is used. When elastic networks are designed, no such reference structure is available, and therefore, an initial random elastic network structure is generated at the beginning of each design procedure. It is obtained by taking a chain of beads which are connected by springs and randomly folding it in the three-dimensional space. Certain spatial constraints are typically imposed to avoid bizarre configurations. After the folding is completed, additional spring connections between all non-neighboring beads are introduced if the spatial separation between them is below a fixed cut-off distance. Thus, a typical compact bead-spring network is generated. Its structure is deformable, and internal motion can, in principle, be induced by the application of external forces. It should be stressed that, although the networks represent fictitious structures, they are constructed in the same way as protein elastic networks. Similar to protein elastic networks, a uniform stiffness constant was also assumed for all network springs. Furthermore, the appropriate choice of geometrical parameters when constructing the backbone in our networks allowed us to adopt cut-off distance values similar to those used in the anisotropic network models of proteins (i.e., typically 8–10 Å [23]). For further detail about the network construction, we encourage the reader to refer to the original publications.

### 3.2. Probing of Network Dynamics

Next, the dynamical properties of the elastic network can be probed, and its performance analyzed with regard to the prescribed function that must eventually be realized. Here, various methods can be employed depending on what is appropriate given the particular application. While, in the work by Togashi and Mikhailov, probing was limited to linear response approximation by computing the normal modes of the network [9], a more thorough force-probe scheme, in which conformational dynamics is determined in response to external forcing by numerically integrating the equations of motion, may be the preferred option [20]. Although the conformational degrees of freedom in the network are very large, it is convenient to characterize the performance of the network in terms of a single or few relevant numbers which can be stored. In our applications, evolution is actually based on optimization with respect to a single entity, referred to as the performance index. Its meaning is related to that of a cost function, which is typically employed in optimization problems. In one of the applications (allosteric networks), it was defined as a simple function of the spatial coordinates of network beads, whereas, in the design procedure of machine-like networks, it was formulated in terms of the spectral gap between the slowest normal modes. Explanations are provided in the corresponding texts and for further detail, we refer the reader to the original publications.

### 3.3. Evolution Cycle

The evolution relies on sequences of mutations followed by selection which are applied iteratively, starting from the initial random network in order to optimize the network performance towards the particular prescribed function. A mutation consists of a small change in the equilibrium position of a randomly selected bead, which, consequently, alters its distance relative to other beads and, therefore, generally results in a reorganization of spring connections, dictated by the cut-off distance. Hence, a mutation corresponds to a local change in the pattern of bead interactions. The performance of the new network with the mutation is then determined by applying the probing procedure and computing the performance index. The selection of a mutation is based on whether it improves the performance of the network, or not, which is evaluated by comparing the performance index of the network before and with the mutation. A mutation is only accepted if it is favorable, and evolution proceeds with the next sequence applied to the new network. Any unfavorable mutation is rejected, and another mutation is tried instead. While optimization with respect to a single entity during evolution represents the simplest implementation, more complex schemes involving aspects of coevolution can also be considered.

### 3.4. Termination

Once a network with sufficient performance is found, as determined by a threshold for the performance index, the evolution is terminated.

## 4. Model Molecular Machine

As a result of biological evolution, protein machines reveal a modular fold that often consists of several domains which are able to perform simple and ordered relative movements with respect to each other. Such intrinsic mechanical motions are coupled to the chemical enzymatic activity of the machine and become cyclically activated by the process of binding an ATP molecule, its subsequent hydrolysis reaction, and the release of chemical products.

A plethora of studies have provided evidence that functional mechanochemical motions in protein machines can be remarkably well described by a single, or a few, slow collective normal modes of the corresponding elastic network (e.g., [6,7,8]). As an important consequence, considerable understanding of the operation mechanism can be obtained, even when chemical aspects are neglected and purely mechanical descriptions aimed at identifying pre-existing motions encoded in a coarse-grained representation of the folded protein structure are employed.

**Figure 2:**
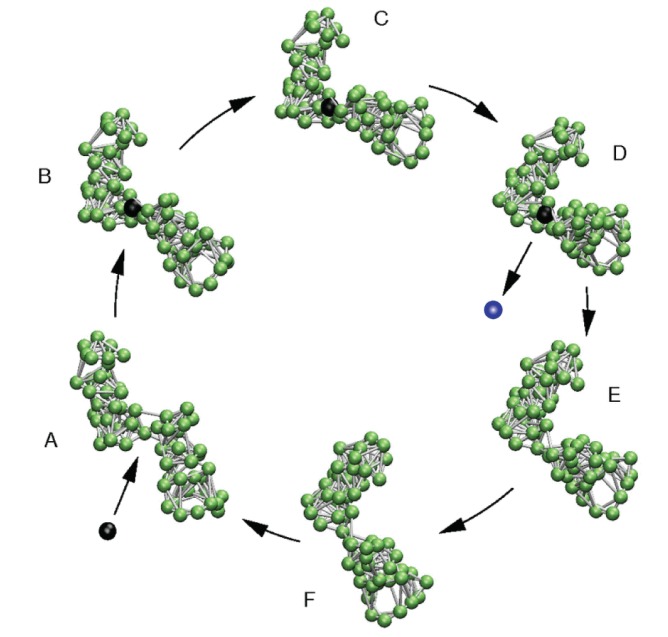
Model molecular machine. A single operation cycle is shown. In the open conformation (A), a substrate ligand bead can bind and trigger the soft hinge motion between the domains (B–D). In the conformation corresponding to the steady state of the network–ligand complex (D), the ligand becomes converted into the product, and after its release, motions which bring back the network to its initial free conformation take place (D–F and A). Figure taken from Reference [24] (slightly modified).

Based on this knowledge, Togashi and Mikhailov proposed a structurally resolved mechanical model of a protein machine in 2007 [9]. Similar to coarse-grained representations of real proteins, it was also constructed as an elastic network and consisted of two connected domains. Then, evolutionary optimization was applied to design a network structure with a single dominant mode in its spectrum, which, as such, should determine conformational relaxation. As a result, the designed network indeed exhibited soft and ordered hinge-bending motions of the two domains. To establish the model machine, this intrinsic motion was excited by modeling the binding and unbinding of a fictitious ligand bead in the flexible hinge region. A single operation cycle of this machine is illustrated in Figure 2 and proceeds as follows: (A) A ligand-mimicking bead (the substrate bead) can bind inside the flexible hinge and connect to some specific network beads there via additional springs. These springs are initially loaded with strain. Hence, after ligand binding, forces are first acting near the binding site and generate motions of network beads there (B). After some time, the soft motion bringing the domains together is triggered (C), and eventually, the new steady state of the network–ligand complex is reached (D). In this state, the ligand (after its conversion to the product) can unbind, after which the network returns to its original conformation (D–F and A). The next ligand-induced cycle is initiated by the binding of another ligand.

The proposed machine mimics the principal operation mechanism of a real protein machine. It is powered by the energy that comes with ligands, which is converted into cyclic, ordered, large-amplitude motions. In the realm of protein machines, the binding ligand would correspond to an ATP molecule, and the ejected different ligand would resemble the post-hydrolysis products, ADP and phosphate. Togashi and Mikhailov also demonstrated that the machine operation is robust, even in the presence of fluctuations.

This machine has been applied in previous studies as a structurally resolved dynamical model. The effect of the solvent on machine operation has been analyzed by considering their hydrodynamical coupling [24]. It has also been employed to investigate the consequences of active molecular machines in membranes, and it has been shown that their cyclic conformational changes induce hydrodynamic lipid flows which affect interactions between active inclusions in biological membranes [25].

We recently used this machine to construct a model system of a molecular motor [22], and we review this study next.

## 5. Model Molecular Motor

Molecular motors are considered to be the class of protein machines that utilize ATP-dependent conformational changes to exert a force on another protein, and thus, perform mechanical work [26,27]. The most prominent examples are probably the cytoskeletal motors, i.e., myosins, kinesins, and dynein, which are able to translate their cyclic internal motions into processive directional locomotion along linear filament tracks, such as actin filaments and microtubules.

**Figure 3:**
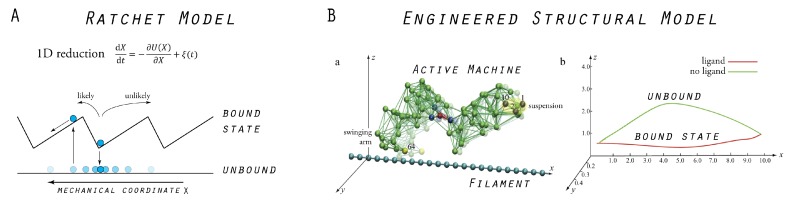
Models of molecular motors: (**A**) Traditional ratchet model in which the motor dynamics is drastically reduced to the problem of a point-like particle moving in a one-dimensional sawtooth potential, or being exposed to diffusion in its absence, respectively. (**B**) Our model molecular motor, consisting of the designed elastic network machine and a rod-like filament. In the presence of a ligand, the machine moves near the filament (and can bind to it), while, after ligand removal, it moves farther away (and is unbound from the filament), as illustrated by the trace of the motor’s swinging arm relative to the filament axis (right figure). Ratcheting transport is thus naturally implemented. Images in (**B**) are adapted from Reference [22] (slightly modified).

Traditionally, and particularly in the community of physicists, the behavior of protein motors is described by a variety of ratchet models, which all have in common the reduction of motor propagation to the stochastic motion of a point-like particle in a one-dimensional potential (see, e.g., [28,29,30]). As a drastic simplification, the motor and the filament structure are not at all resolved, and their repeated interactions are purely phenomenologically accounted for by imposing a periodic potential which has an asymmetric sawtooth shape (see Figure 3). The model typically incorporates two different states of the motor. One corresponds to the bound state of the motor–filament system and is represented by the confinement of the particle around a minimum in the potential, and the other resembles the unbound state which is represented by the particle being exposed to diffusion. Within this model, unidirectional motor translocation is described by switching between the two states, which is achieved, for instance, by repeatedly switching the potential on and off, which results in the particle being effectively transported in the prescribed direction, defined by the potential tilt. Comprehensive reviews of ratchets as models of biological motors are available, and we restrict ourselves to a discussion of how our model of a molecular motor relates to ratchet models.

Our model of a molecular motor is composed of the cyclically operating machine described in the previous section and a rod-like filament. The machine’s structure is immobilized at one site and the filament is placed below the other domain, the swinging arm. After ligand binding to the machine and subsequent transition to the closed conformation, the tip of this arm moves in close proximity and almost parallel to the filament. In contrast, after ligand release occurs and the machine returns to its open conformation, the arm moves along a path farther away from the filament (see Figure 3). This asymmetry was employed to introduce distance-dependent attractive interactions between the machine’s swinging arm and the filament. Bonds between the arm and the filament can form if they are close one another and become unformed at larger distances. Hence, a ratchet mechanism of filament transport by the machine was naturally implemented. In the presence of the ligand, the machine can bind to the filament and perform a power stroke (the force-producing step), under which its conformational motion is translated into dragging of the filament. The filament is held and moves until the ligand becomes converted into the product. After product release, the swinging arm moves away from the filament, the connecting bonds become broken, and during the second part of the cycle, the machine returns to its equilibrium open conformation, remaining detached from the filament.

The established machine–filament system mimics the principal function that is commonly associated with biological molecular motors. In Figure 3, we schematically show how our model motor compares to traditional ratchet models. Through extensive numerical simulations, we analyzed the model motor and identified different regimes which characterize its mechanism of operation.

### 5.1. Tight Coupling Mechanism—Power Stroke Ratcheting Mode

The motor behavior was studied as the level of thermal fluctuations increased, while, at the same time, the coupling strength of the motor to the filament was always maintained. The weak fluctuation regime obtained at low temperatures was characterized by strong coupling of the motor to the filament. After ligand binding, the machine was able to tightly grasp the filament and execute the power stroke under which the filament followed the motion of the machine and was transported by a clear step. After product release, the machine and filament performed independent fluctuations. Through this operation regime, the motor can steadily transport the filament by clear steps, one step per ligand-induced cycle, although the size of steps is subject to variation due to the stochastic nature of the interactions between ligands and the machine. The motor behavior in this regime resembles the tight coupling mechanism of protein motors. We elaborate further on commonly debated operation mechanisms for protein motors in Section 7.

The action of the motor in this regime can be qualitatively related to the ratchet models. The level of thermal fluctuations is much lower than the maximum barrier height of the interaction potential between the machine and the filament. Therefore, each time a ligand is bound to the machine, the filament gets locked in a traveling trough of the potential and moves together with it. However, in the absence of a ligand, the swinging arm of the machine is separated too far from the filament and the interaction potential vanishes. Hence, the motor operates similar to a deterministic ratchet (see Section 7).

### 5.2. Loose Coupling Mechanism—Brownian Ratchet Mode

In the case of strong thermal fluctuations, the behavior of the model motor was very different and was characterized by weak coupling of the motor to the filament. The machine itself was still able to robustly perform its internal ligand-induced conformational cycles. However, the swinging arm could never firmly grasp the filament, but instead, slid against it when bonds between the two frequently became formed as well as unformed. No clear steps of filament transport were identified in this regime and, essentially, the motor was shown to only weakly affect the intrinsic Brownian motion of the filament. Nonetheless, this perturbation could still generate some net driving force acting on the filament, resulting, on average, in its directional transport. The motor behavior in this regime resembles the loose coupling mechanism of protein motors (see Section 7).

This mode of operation can also be related to the ratchet description. In this regime, the thermal energy is always higher than the barrier of the interaction potential between the machine and the filament. Therefore, no steady transport within a single cycle can be performed by the motor, and yet, effectively, unidirectional filament motion is accomplished. The dynamics of the motor–filament system is dominated by strong fluctuations, but, since the motor is operating under non-equilibrium conditions, work can effectively be extracted from its internal cycling. In that sense, the motor operation resembles a Brownian ratchet (see Section 7).

### 5.3. Designed Motor as a Model System

**Figure 4:**
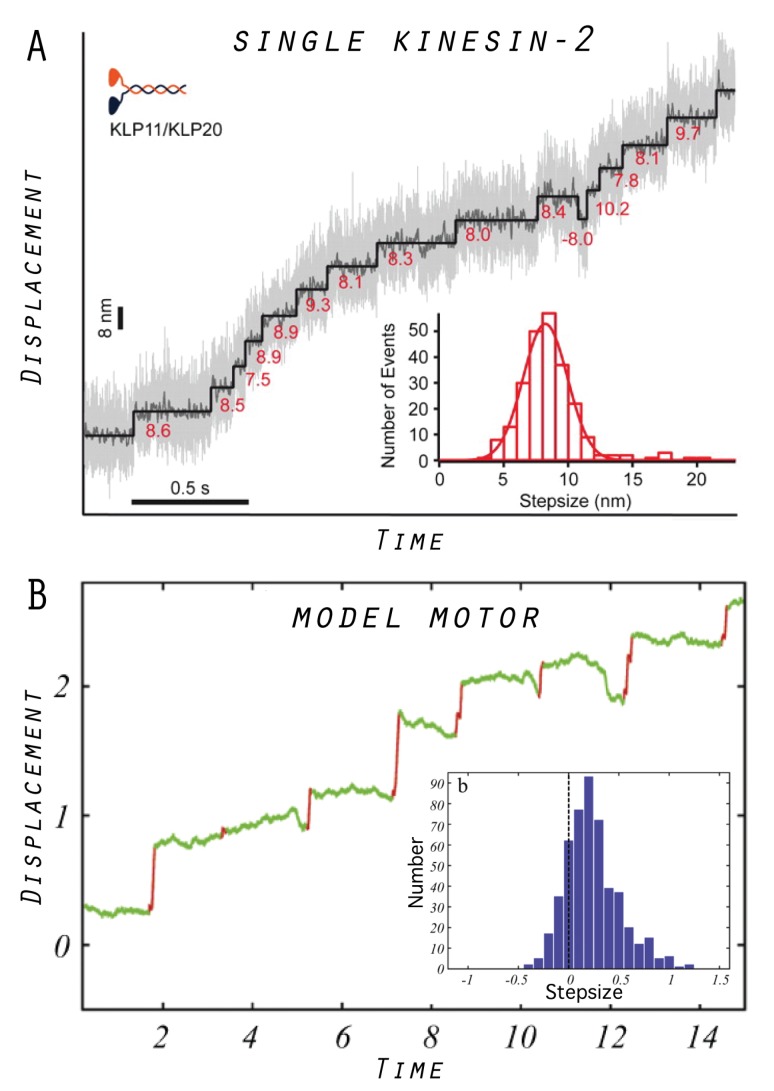
Examples of motor operation: (**A**) Stepwise processive transport by a kinesin-2 motor observed in single-molecule assays under saturating ATP concentrations. The stepsize distribution is also shown. The figure is taken from Reference [31]. (**B**) An example trace of filament transport by the designed in silico model motor, and the stepsize distribution obtained from the analysis of 1000 operation cycles (adapted from Reference [22] with modifications).

In our model, the same device can operate as either a tightly coupled or a loosely coupled motor, the scenarios which represent the commonly debated working mechanisms of protein motors in the scientific community (see Section 7). Furthermore, there is a continuous transition between the regimes which proceeds through an intermittent operation mode. For this intermittent regime of the model motor, a characteristic trajectory of filament transport is shown in Figure 4, together with the distribution of transport steps obtained from the statistical analysis of 1000 operation cycles. For a qualitative comparison with a real protein motor, we also show similar data obtained from a single-molecule experiment of a specific kinesin-2 motor system [31]. The trajectories look similar, showing the step-like transport by both motors, and, in both cases, a significant dispersion in the distribution of transport steps is present as a result of thermal fluctuations.

In experiments of molecular motors, the operation under external load, represented, e.g., by some cargo attached to the motor, is very important. Such a scenario has also been implemented for the proposed model motor by considering its operation under the action of external forces and analyzing its behavior when approaching the stall regime.

Our engineered artificial motor still represents a reduced model and is, in all aspects, much less sophisticated than a real biological motor. Nonetheless, it can qualitatively reproduce characteristic operation cycles and allows different possible operation regimes to be explored and the effect of thermal fluctuations to be examined in a structurally resolved way. We discuss the advantages of our designed motor as a new type of model system for protein motors in Section 7.

## 6. Models of Allosteric Protein Machines

Allosteric communication represents one of the most important and sophisticated phenomena underlying protein operation. Its investigation has, therefore, been the subject of a tremendous body of research in the past, and still generates enormous attention in current research. While the majority of studies have been dedicated to understanding the allosteric effects of a particular protein given its specific folded structure, the ubiquity of allostery among all domains of macromolecular functioning raises more fundamental and general questions; for example, can we identify generic design principles of allosteric structures or functional signatures which are essential for allosteric phenomena, or unique to them?

Motivated by such questions, we considered the design of elastic networks as a novel approach towards providing a general and simplified, yet structurally resolved, mechanical model of allosteric coupling in complex structures.

Consistent with the prospects of elastic network dynamics, the modeling idea was inspired by the mechanical perspective of allostery, according to which, intramolecular communication is a consequence of a protein quake [32,33]. Mediated by a network of interacting particles, local conformational motions, initiated, e.g., upon ligand binding, propagate through the protein structure and generate a functional change in the conformation of a distant site.

We have, therefore, designed elastic networks which, as the principal element of allosteric operation, encode optimal long-range communication between two distant sites. In those special network structures, conformational motions triggered at one site generate significant motion at a remote site. First, the design process, which is based on the evolutionary optimization described in the previous section, is summarized. Then, we put emphasis on how the analysis of dynamical properties helped with the understanding of the mechanistic underpinnings of allosteric communication in the designed prototype structures. The relationship between the constructed model systems and the realm of real allosteric proteins is also discussed.

### 6.1. Design of Allosteric Network Structures

The initial random elastic structure was constructed as a network consisting of 200 beads. In this structure, two pockets were chosen, each formed by two beads which were located on opposite sites. The allosteric performance of an elastic network was always probed according to the cause-and-effect causality. First, binding of a ligand to one site was initiated (the cause); second, conformational motions were followed as they propagated through the entire network structure; and, third, the response generated at the other site was detected (the effect).

In the random network, no allosteric response could be detected. The arbitrary initial configuration of beads and connections between them did not allow the structure to conduct ligand-induced motions through it. It therefore represents an *allosteric insulator*, and we employed evolutionary optimization to design structures to encode long-range communication, i.e., networks in which the ligand event at one site is coupled to an enhanced response at the remote site. The evolution cycle consisted of sequences of a mutation followed by probing of the allosteric response and selection, which were iteratively applied to improve the allosteric performance of the network. To score the allosteric response, only the size change of the remote pocket was evaluated and used as the performance index to be optimized during evolution. Two prototype networks which revealed optimized allosteric communication were designed. The first encodes symmetric allosteric coupling, i.e., ligand-induced closing of the first pocket results in closing of the remote second pocket. The second network encodes asymmetric coupling, where the allosteric response consists of opening the second pocket. The design processes both started from the same initial random elastic network, but the two strains of evolution proceeded independently.

### 6.2. Prototype Allosteric Structures

After obtaining the two functional networks, we focused on understanding the mechanisms by which each of them is able to establish the allosteric communication which is encoded in their structure as a result of evolution. Apparently, the long-range coupling of large-amplitude motions localized in the two remote pockets is established by conformational motions which spread from one site to the other. To characterize this process, we analyzed the mechanical strain of the network which corresponds to the deformation of elastic springs.

**Figure 5:**
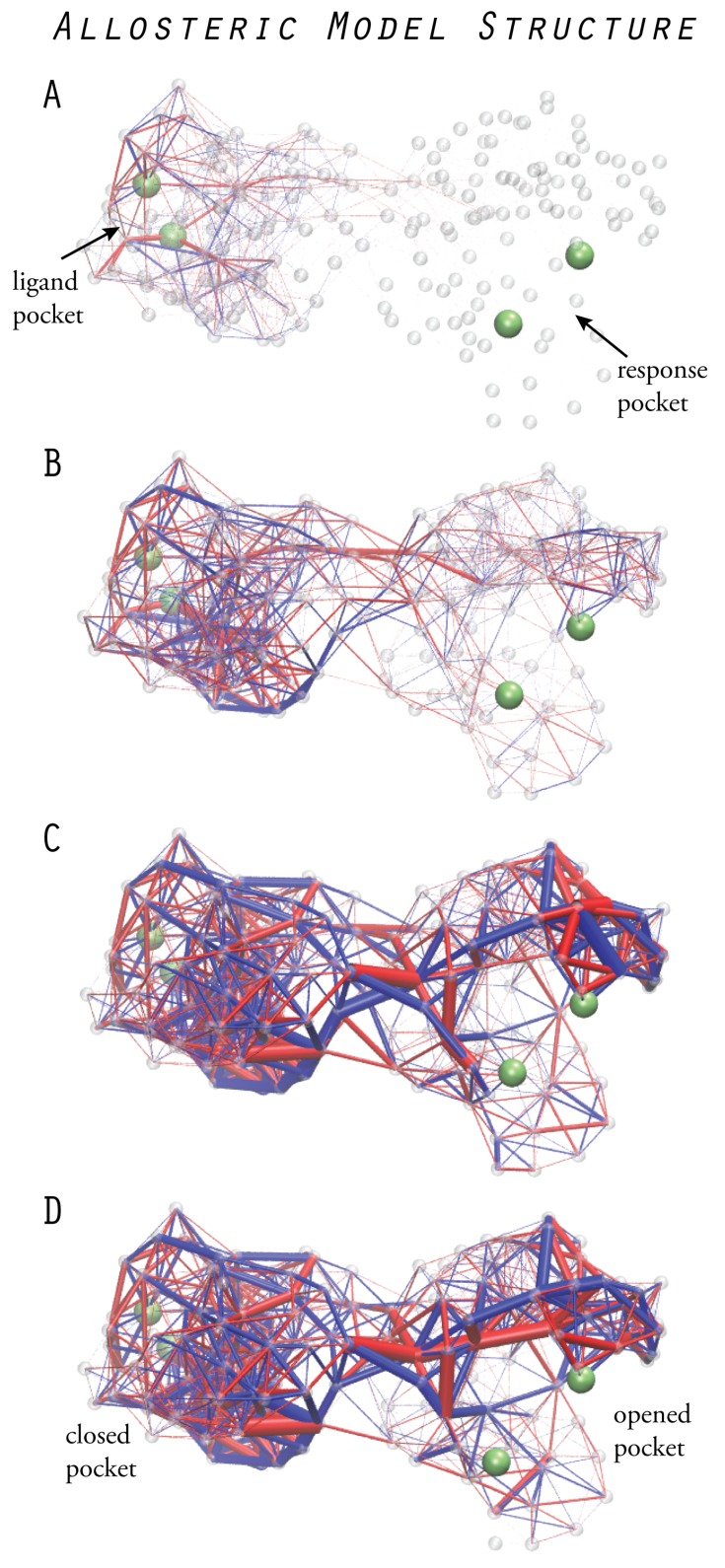
Designed allosteric structure. Prototype elastic network structure with an asymmetric allosteric response. The propagation of strain, shown at different moments of time after ligand binding (**A**–**D**), reveals communication pathways which couple the dynamics of the remote pockets (snapshots adopted from Reference [20]).

In both designed structures, we could follow the temporal order of strain becoming propagated through the structure after ligand binding (see Figure 5). First, only links in the vicinity of the ligand pocket became strained. During the second stage, strain populated also links in the interface region between the two domains, and finally, a set of links in the second domain including some close to the second pocket became occupied with strain, eventually generating the large-amplitude motion there.

A remarkable observation was made for both designed structures. From all network links, only a subset was found to be significantly strained, and many of them, in particular, those in the interface region and inside the second domain, showed negligible strain. A further analysis, considering only those links which were most strained, allowed us to identify the critical communication patterns (for details, we refer to the original paper [20]). In the designed networks, strain propagation proceeds through well-defined pathways, and highly strained links even form simple chains which connect the two remote pockets. They can be viewed as a primitive channeling system through which strain flows from one site to the other. Figure 5 shows strain propagation in one designed network structure.

The analysis of strain provides a mechanistic understanding of allosteric communication in the designed networks. Moreover, this directly relates the existence of definite communication chains and pathways as functional signatures which emerge during evolution. In random networks, such properties are absent, and they are generally allosterically inactive. During design, the identified functional signatures emerge under evolutionary pressure to improve and, eventually, optimize allosteric communication in the network structures.

The structure of real allosteric proteins is obviously the result of natural biological evolution which was aimed at perfecting communication between different functional sites in the molecular structure. Through nuclear magnetic resonance experiments, it has, indeed, become possible to provide evidence for the existence of communication pathways in allosteric proteins [34,35]. They can be identified as a set of physically interacting amino acid residues which span the region between functional sites and thus, link their activity. In many structure-based computational studies, including investigations of protein elastic networks [36,37,38,39,40,41], functional conformational motions in allosteric proteins have been resolved and communication pathways have been identified based on physical methods.

It is a truly remarkable result that the elastic network structures, designed under the framework of our minimal evolution scheme, reveal similar functional properties in terms of communication pathways which establish allosteric coupling. For the purpose of demonstration, we applied the modeling approach of ligand binding, together with the analysis of strain propagation and the methods to quantify communication pathways, and investigated allosteric coupling in a real protein system. For the myosin-V molecular motor domain, the conformational motions from the nucleotide-free to the ATP-bound state were followed [20]. This transition involves allosteric coupling to the remote actin cleft, and its expected opening in response to ligand binding to the ATP pocket was reproduced very well in our dynamical simulations. By following conformational dynamics after ATP binding, we were able to visualize strain propagation into the actin binding cleft and extract the communication pathways which couple motions in the two remote functional regions. Notably, they coincided with well-known residue motifs which have previously been identified to be critical for chemomechanical coupling in the myosin V motor.

**Figure 6:**
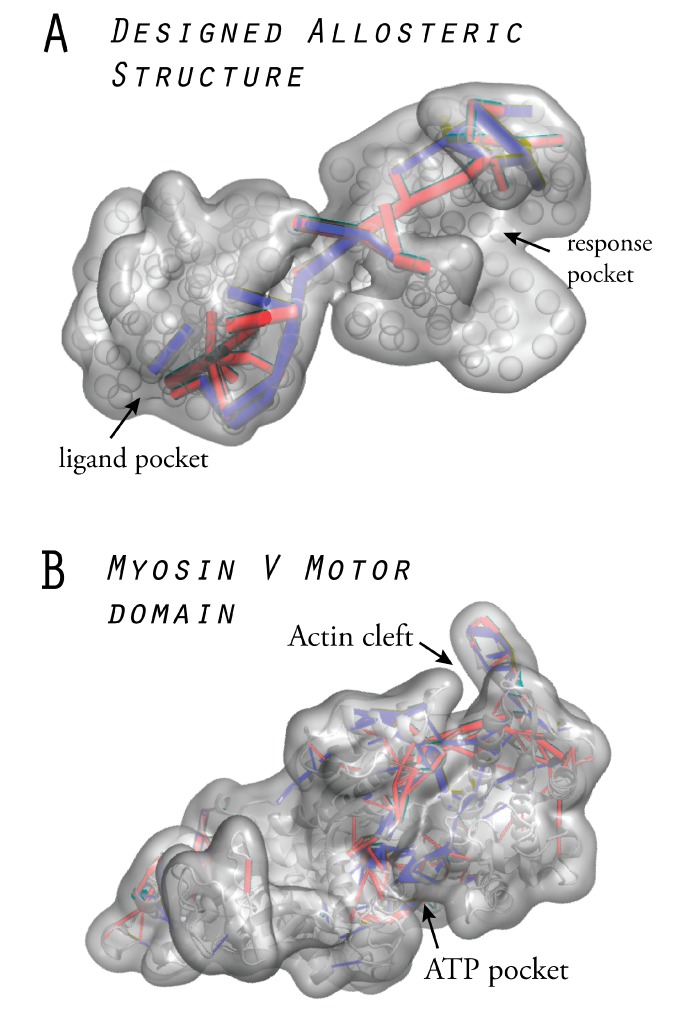
Allosteric structures: (**A**) A designed elastic network which encodes optimal long-range communication between two distant pockets represents a fictitious allosteric protein structure. (**B**) The myosin-V motor domain in which allosteric regulation between the ATP-binding pocket and the actin interaction cleft is essential for mechanochemical coupling. Both structures are shown in the surface representation, and the identified communication pathways are highlighted. Results from Reference [20] are incorporated into both figures.

Figure 6 displays a designed allosteric structure obtained from our computational evolution model, together with the naturally evolved myosin V motor domain. In both structures, the identified communication pathways are highlighted. In fact, the two designed network architectures can be regarded as coarse-grained representations of fictitious, allosterically functional protein structures. We elaborate on such aspects further in Section 7.

### 6.3. Mechanical Networks with Allosteric Interactions

Thus far, we have reviewed the design of elastic networks with the motivation coming from the coarse-grained modeling of protein dynamics. Other groups have reported exciting applications that emphasize different viewpoints. Rocks et al. implemented a computational strategy to tune mechanical networks towards developing allosteric responses [18]. This was achieved by pruning bonds in a random elastic network. Theoretical considerations have been put into practice by fabricating macroscopic 2D and 3D metamaterials which exhibit specific allosteric responses. The effect of bond removal on the elastic properties of spring networks has also been investigated in the context of material science [42]. It has been further demonstrated that tuning based on adding or removing links can be employed to generate networks which display multifunctional responses [43]. Yan et al. considered 2D on-lattice models which evolve under the swapping of springs to accomplish an allosteric task [19], and deduced design and physical aspects in emerging functional networks. Two-dimensional elastic networks were also used by Dutta et al. to design structures with dynamics reminiscent of allosteric effects in proteins [21]. In their model, physical interactions between network beads were described in terms of the HP (hydrophobic-polar) model, and the emergence of functionality was found to be related to the existence of collective modes that describe the propagation of motion.

The commonality amongst those studies is that network templates are represented as 2D systems of connected springs. It has been demonstrated that multiple methods of manipulating interactions are applicable to successfully design functionality in networks (bond removal, bond swapping, and stiffness variation). When they are viewed in the context of protein models, the employed descriptions have the character of toy models due to their more abstract nature. In that regard, we also want to mention the earlier work by Borovinskiy and Grosberg, in which *toy proteins* were designed within the framework of polymer lattice models [44]. Furthermore, in all studies, the allosteric dynamics of a network was formulated within the linear response theory. Although this approximates the generally nonlinear dynamics of elastic networks, it was shown to be reasonable within the considered framework.

There are several aspects for which our approach differs from the above-mentioned implementations. Most notably, we designed structures which are consistent with the elastic network models used to investigate dynamics of real proteins. Specifically, they consisted of a backbone folded in the three-dimensional space; the network connectivity was determined according to a fixed cut-off distance; changes in network connections subsequent to local structural changes (mutations) were performed in accordance with the cut-off rule; and a uniform spring stiffness was assumed. Therefore, the designed model structures can be viewed as coarse-grained representations of fictitious protein structures. As an important consequence, a comparison of their architectures and functional properties to actual protein elastic networks is reasonable, and the first steps in that direction have been undertaken by investigating pathways of allosteric communication. It should be stressed that protein elastic networks are not just toy models, and their explanatory power is widely appreciated in the context of protein modeling.

As a further distinction to all other works, we have treated elastic networks as dynamical systems by considering their complex dynamics beyond the linear response limitation (normal mode analysis). Though the possible contribution of nonlinear effects to the allosteric dynamics of the networks has not yet been elucidated, they are generally included in our models. Our dynamical simulations enabled us to resolve the temporal order of events which establish long-range allosteric coupling inside the networks. In fact, the analysis of strain propagation allowed us to identify pathways and communication chains as functional signatures in our designed networks.

## 7. Discussion

Recently, the design of mechanical networks and control of their functional properties has become highly popular, with applications ranging from material sciences to biophysical problems. It is too early to give a comprehensive review of the published work, and in this topical review, we decided to emphasize the work which was motivated by our studies of protein dynamics employing coarse-grained elastic network models. While we focused mainly on reviewing our own contributions to this field and provided only a brief discussion of related work, we hope that the presented perspective will still be of value for researchers in the field and stimulate further discussions. We next give some concluding remarks and discuss the advantages of the designed networks as model systems of complex protein machines.

### 7.1. Evolution Model: Autonomously Learning Structures and Dimensionality Reduction

To design functional network structures, we developed a strategy of evolution which consists of cycles of mutations followed by selection. Though the network architectures do not correspond to real existing protein structures, our evolution model is related to the biological evolution of proteins. In our model, a single mutation can only locally and slightly change the equilibrium network structure (and hence alter the local pattern of interactions), while the global architecture remains unaffected. In the realm of protein evolution, this corresponds to a point mutation in the genotype upon which the folded structure is maintained, except for small changes localized around the mutation spot caused by side chain variations.

An important aspect which should be stressed is that this evolution model can be regarded as an autonomous learning process for the network structures. During evolution cycles, the evolutionary pressure corresponds to the optimization of a single observable. In the case of the model machine, the spectral gap is maximized, and for the design of allosteric structures, the pressure solely magnifies the response in the remote pocket. Other than optimizing the appropriately chosen observable, no other requirements are imposed, and while, during evolution, the structures improve their performances towards their desired function, their underlying architectures and dynamical properties emerge autonomously.

Another aspect that we want to discuss is that the design of our protein-mimicking model systems through the evolution scheme represents a demonstration of dimensionality reduction, which has been previously associated with functional specialization in models of protein evolution (e.g., [45]). In the case of the designed model machine, the reduction of dynamics is achieved automatically, imposed by the requirement that slow conformational dynamics becomes dominated by a single normal mode. However, when network structures with perfect allosteric communication were designed, the full nonlinear elastic dynamics were always considered. However, it turns out that as evolution progresses, a single soft mode (or a few of them) emerges in the spectra of the evolving networks which adequately described the allosteric transition (to be published). Therefore, the allosteric communication which has emerged as a result of evolution effectively corresponds to low-dimensional (even one-dimensional) dynamical processes in the networks. Recently, those observations were reported for the design of allosteric effects in 2D elastic networks [21,46].

Related to those aspects, we remark that, in a number of previous studies, we have shown that the relaxation dynamics in the elastic networks of protein machines and motors is effectively reduced to low-dimensional attractive bundles in the conformation space, and corresponds to well-defined, ordered domain motions [9,10,11,12,16]. We concluded that this property is likely the result of biological evolution, and it ensures that protein machines and motors can robustly execute the same intrinsic conformational motions in each operation cycle.

### 7.2. Structurally Resolved Model Systems of Protein Machinery

Our studies have demonstrated that elastic networks can be designed as fictitious protein structures emulating their operation. As such, they may provide a novel class of model systems which allow the design and functional principles of their real biological counterparts to be revealed and better understood in a structurally resolved way.

Operating at the nanoscale, protein motors are subject to thermal fluctuations and their behavior is of stochastic nature. Two distinct concepts are debated in the community to describe their mechanochemical activity. The *tight coupling* mechanism involves a strong correlation of ATP-related conformational cycles and the force-producing power stroke which would result in regular transport steps. This type of mechanism is employed by many cytoskeletal motors, e.g., myosin V [47,48], as the strategy to transport cargo over long distances, since it ensures consistent step-by-step movement. In contrast, the *loose coupling* mechanism is characterized by weak coupling of internal motor motion and movement outputs [49]. It has been experimentally demonstrated to be the underlying mechanism of muscle contraction by the actin–myosin II motor system [48,50]. Both scenarios of motor operation are realized within the oversimplified ratchet descriptions, where the latter is referred to as *Brownian ratchets*, while the former resembles *deterministic ratchet* behavior. (The term *deterministic ratchet* is not commonly used within the motor community. There is no determinism in the presence of thermal fluctuations).

Our model of a protein motor was engineered to emulate the principal operation on any known molecular motor—a machine which consumes energy and converts it into mechanical work and movement. The machine is an elastic network structure which, as a result of the applied evolution, can perform ordered and robust domain motions. Conformational motions in this model machine are cyclically initiated and powered by the energy coming with ligands, similar to the ATP-powered activity of a real protein machine. During its operation cycles, the machine can interact with a filament and transport it. When the machine has a ligand bound, its swinging arm moves close to the filament and binds to it. After the ligand is released from the machine, the arm moves farther away from the filament and is detached. Therefore, in our model motor, a ratchet mechanism is naturally implemented as a result of the internal operation of the motor system and is not externally imposed. It is the device’s key element, allowing to translate the cyclic internal conformational motions of the ligand-powered machine into the directional transport of the filament.

The constructed motor can qualitatively emulate the operation of a real protein motor. As a model system, its advantage over traditional ratchet models is that it allows the functional aspects of motor operation to be investigated in a structurally resolved way. For example, the motor operation in various parameter regimes can be explored in dynamical simulations, and its behavior can be explained by the effects of fluctuations on motions of the swinging arm and how they affect interactions with the filament. A fluctuation analysis of our model system showed that the same motor incorporates the *tight coupling* mechanism and can perform clear step-like transport through the generation of power strokes, as well as the *loose coupling* or Brownian ratchet mechanism when motor dynamics is dominated by fluctuations, and yet, on average, effective transport is achieved. Our model allows the transition between both regimes and continuous changes in the motor behavior to be investigated. Furthermore, motor operation under external loads simulating the effect of cargo can be analyzed. Finally, an important advantage of our model system is that thousands of functional cycles can be monitored in the simulations, enabling us to perform a statistical analysis of motor operation. It can be said with almost certainty that anything like that will never become possible in atomistic-level simulations of protein motors.

Allostery represents the most ubiquitous and sophisticated phenomena in protein dynamics. Traditionally described by the MWC (Monod–Wyman–Changeux) and KNF (Koshland–Némethy-Filmer) models [51,52], which were formulated more than 50 years ago in the absence of any structural data and were therefore of purely phenomenological nature, the allosteric effects in proteins are nowadays typically investigated in structure-based simulations at different resolution levels. The fact that allosteric effects represent information which is universally encoded across highly diverse protein structures motivated us to design fictitious protein structures which, as the principal element of allosteric coupling, encode optimized long-range communication between distant sites. As a remarkable result, the networks designed in our evolution scheme have revealed functional properties which are similar to those found in real proteins, i.e., allosteric communication is established through well-defined pathways and simple interaction chains which connect the distant sites. Another communality with proteins is that, in the designed structures, a single critical mutation can inhibit allosteric coupling. The advantage of our designed network structures as model systems is that the temporal order of events which underlie allosteric effects is resolved. Beginning with localized ligand binding, the propagation of conformational changes and elastic strains through the structures, which eventually generate motion in the remote pocket, is monitored. This model naturally incorporates causality as the leading principle of allosteric coupling. Its explanatory power is therefore richer than most other studies, in which allosteric communication is often investigated based on the computation of normal modes and the analysis of correlated residue motions.

To conclude, our studies demonstrate that the design of elastic networks with protein-inspired functions may allow a promising and elegant journey towards better understanding complexity in protein machines.

Recently, much progress has been made in the construction of artificial proteins with a given structural fold (see, e.g., [53]). Our model studies are not yet sophisticated enough to address questions related to the possible realization of a concrete molecular system which would show the designed responses. Nonetheless, we hope that our studies help to formulate the constraints and design principles which are important when the engineering of bio-molecular machines and motors is considered in the future. It may also be possible to combine the developed approach with computational methods of structure predictions and experiments.

## References

[1] Alberts B. (1998). The cell as a collection of protein machines: Preparing the next generation of molecular biologists. Cell.

[2] Mikhailov A.S., Ertl G. (2017). Molecular machines. Chemical Complexity, Self-Organization Processes in Molecular Systems.

[3] Tirion M.M. (1996). Large amplitude elastic motions in proteins from a single-parameter, atomic analysis. Phys. Rev. Lett..

[4] Bahar I., Atilgan A.R., Erman B. (1997). Direct evaluation of thermal fluctuations in proteins using a single-parameter harmonic potential. Fold. Des..

[5] Haliloglu T., Bahar I., Erman B. (1997). Gaussian dynamics of folded proteins. Phys. Rev. Lett..

[6] Tama F., Sanejouand Y.H. (2001). Conformational change of proteins arising from normal mode calculations. Prot. Eng..

[7] Zheng W., Doniach S. (2003). A comparative study of motor-protein motions by using a simple elastic-network model. Proc. Natl. Acad. Sci. USA.

[8] Yang L., Song G., Jernigan R.L. (2007). How well can we understand large-scale protein motions using normal modes of elastic networks. Biophys. J..

[9] Togashi Y., Mikhailov A.S. (2007). Nonlinear relaxation dynamics in elastic networks and design principles of molecular machines. Proc. Natl. Acad. Sci. USA.

[10] Flechsig H., Mikhailov A.S. (2010). Tracing entire operation cycles of molecular motor hepatitis C virus helicase in structurally resolved dynamical simulations. Proc. Natl. Acad. Sci. USA.

[11] Togashi Y., Yanagida T., Mikhailov A.S. (2010). Nonlinearity of mechanochemical motions in motor proteins. PLoS Comput. Biol..

[12] Flechsig H., Popp D., Mikhailov A.S. (2011). In silico investigation of conformational motions in superfamily 2 helicase proteins. PLoS ONE.

[13] Düttmann M., Togashi Y., Yanagida T., Mikhailov A.S. (2012). Myosin-V as a mechanical sensor: An elastic network study. Biophys. J..

[14] Flechsig H. (2014). Computational biology approach to uncover hepatitis C virus helicase operation. World J. Gastroenterol..

[15] Flechsig H. (2014). TALEs from a spring–superelasticity of Tal effector protein structures. PLoS ONE.

[16] Flechsig H. (2016). Nucleotide-induced conformational dynamics in ABC transporters from structure-based coarse grained modeling. Front. Phys..

[17] Dai L., Flechsig H., Yu J. (2017). Deciphering intrinsic inter-subunit couplings that lead to sequential hydrolysis of F1-ATPase ring. Biophys. J..

[18] Rocks J.W., Pashine N., Bischofberger I., Goodrich C.P., Liu A.J., Nagel S.R. (2017). Designing allostery-inspired response in mechanical networks. Proc. Natl. Acad. Sci. USA.

[19] Yan L., Ravasio R., Brito C., Wyart M. (2017). Architecture and coevolution of allosteric materials. Proc. Natl. Acad. Sci. USA.

[20] Flechsig H. (2017). Design of elastic networks with evolutionary optimised long-range communication as mechanical models of allosteric proteins. Biophys. J..

[21] Dutta S., Eckmann J.-P., Libchaber A., Tlusty T. (2018). Green function of correlated genes in a minimal mechanical model of protein evolution. Proc. Natl. Acad. Sci. USA.

[22] Sarkar A., Flechsig H., Mikhailov A.S. (2016). Towards synthetic molecular motors: A model elastic-network study. New J. Phys..

[23] Atilgan C., Gerek Z.N., Ozkan S.B., Atilgan A.R. (2010). Manipulation of conformational change in proteins by single-residue perturbations. Biophys. J..

[24] Cressman A., Togashi Y., Mikhailov A.S., Kapral R. (2008). Mesoscale modeling of molecular machines: Cyclic dynamics and hydrodynamical fluctuations. Phys. Rev. E.

[25] Huang M.J., Kapral R., Mikhailov A.S., Chen H.-Y. (2013). Coarse-grain simulations of active molecular machines in lipid bilayers. J. Chem. Phys..

[26] Spudich J.A. (1994). How molecular motors work. Nature.

[27] Vale R.D., Milligan R.A. (2000). The way things move: Looking under the hood of molecular motor proteins. Science.

[28] Cordova N.J., Ermentrout B., Oster G. (1992). Dynamics of single-motor molecules: The thermal ratchet model. Proc. Natl. Acad. Sci. USA.

[29] Astumian R.D., Bier M. (1994). Fluctuation driven ratchets: Molecular motors. Phys. Rev. Lett..

[30] Jülicher F., Ajdari A., Prost J. (1997). Modeling molecular motors. Rev. Mod. Phys..

[31] Brunnbauer M., Mueller-Planitz F., Kösem S., Hieu Ho T., Dombi R., Gebhardt J.C.M., Rief M., Ökten Z. (2010). Regulation of a heterodimeric kinesin-2 through an unprocessive motor domain that is turned processive by its partner. Proc. Natl. Acad. Sci. USA.

[32] Ansari A., Berendzen J., Bowne S.F., Frauenfelder H., Iben I.E., Sauke T.B., Shyamsunder E., Young R.D. (1985). Protein states and proteinquakes. Proc. Natl. Acad. Sci. USA.

[33] Tsai C.-J., Nussinov R. (2014). A unified view of how allostery works. PLoS Comput. Biol..

[34] Brüschweiler S., Schanda P., Kloiber K., Brutscher B., Kontaxis G., Konrat R., Trollinger M. (2009). Direct observation of the dynamic process underlying allosteric signal transmission. J. Am. Chem. Soc..

[35] Grutsch S., Brüschweiler S., Trollinger M. (2016). NMR methods to study dynamic allostery. PLoS Comput. Biol..

[36] Xu C., Tobi D., Bahar I. (2003). Allosteric changes in protein structure computed by a simple mechanical model: Hemoglobin T-R2 transition. J. Mol. Biol..

[37] Bahar I., Chennubhotla C., Tobi D. (2007). Intrinsic dynamics of enzymes in the unbound state and relation to allosteric regulation. Curr. Opin. Struct. Biol..

[38] Yang Z., Majek P., Bahar I. (2009). Allosteric transitions of supramolecular systems explored by network models: Application to chaperonin GroEL. PLoS Comput. Biol..

[39] Erman B. (2013). A fast approximate method of identifying paths of allosteric communication in proteins. Proteins.

[40] Yao X.-Q., Skjaerven L., Grant B.J. (2016). Rapid characterization of allosteric networks with ensemble normal mode analysis. J. Phys. Chem. B.

[41] Hacisuleyman A., Erman B. (2017). Causality, transfer entropy, and allosteric communication landscapes in proteins with harmonic interactions. Proteins.

[42] Hexner D., Liu A.J., Nagel S.R. (2018). Role of local response in manipulating the elastic properties of disordered solids by bond removal. Soft Matter.

[43] Rocks J.W., Ronellenfitsch H., Liu A.J., Nagel S.R., Katifori E. (2018). The limits of multifunctionality in tunable networks. arXiv.

[44] Borovinskiy A.L., Grosberg A.Y. (2003). Design of toy proteins capable of rearranging conformations in a mechanical fashion. J. Chem. Phys..

[45] Tlusty T., Libchaber A., Eckmann J.-P. (2017). Physical model of the genotype-to-phenotype map of proteins. Phys. Rev. X.

[46] Yan L., Ravasio R., Brito C., Wyart M. (2018). Principles for optimal cooperativity in allosteric materials. Biophys. J..

[47] Sakamoto T., Webb M.R., Forgacs E., White H.D., Sellers J.R. (2008). Direct observation of the mechanochemical coupling in myosin Va during processive movement. Nature.

[48] Nishikawa M., Takagi H., Shibata T., Iwane A.H., Yanagida T. (2008). Fluctuation analysis of mechanochemical coupling depending on the type of biomolecular motors. Phys. Rev. Lett..

[49] Oosawa F. (2000). The loose coupling mechanism in molecular machines of living cells. Genes Cells.

[50] Yanagida T., Arata T., Oosawa F. (1985). Sliding distance of actin filaments induced by a myosin cross-bridge during one ATP hydrolysis cycle. Nature.

[51] Monod J., Wyman J., Changeux J.-P. (1965). On the nature of allosteric transitions: A plausible model. J. Mol. Biol..

[52] Koshland D.E., Nemethy G., Filmer D. (1966). Comparison of experimental binding data and theoretical models in proteins containing subunits. Biochemistry.

[53] Koga N., Tatsumi-Koga R., Liu G., Xiao R., Acton T.B., Montelione G.T., Baker D. (2012). Principles for designing ideal protein structures. Nature.

[54] Humphrey W., Dalke A., Schulten K. (1996). VMD—Visual molecular dynamics. J. Mol. Graph..

